# Differentiation of mucosal lesions in mucocutaneous leishmaniasis and paracoccidioidomycosis

**DOI:** 10.1371/journal.pone.0208208

**Published:** 2018-11-26

**Authors:** Creuza Rachel Vicente, Aloisio Falqueto

**Affiliations:** Department of Social Medicine, Health Sciences Center, Federal University of Espírito Santo, Vitória, Espírito Santo State, Brazil; Universidade Federal da Bahia, BRAZIL

## Abstract

Mucocutaneous leishmaniasis and paracoccidioidomycosis are infectious diseases with similar epidemiological and clinical aspects. Cases of both diseases may manifest similar lesions in the mucosa. Therefore, the determination of distinguishing characteristics for the purpose of differential diagnosis is critical for better management of the diseases. The present study evaluated factors that assist in the differentiation of mucosal lesions between these diseases. This cross-sectional study included data from medical records of 122 cases of mucocutaneous leishmaniasis and 83 cases of paracoccidioidomycosis attended at the university hospital Cassiano Antonio Moraes, located in Vitória, Espírito Santo State, Brazil. Comparison between the diseases included the following variables: sex, age, time of disease evolution, location of the lesion and symptoms. Adults and males were affected by both diseases at higher rates. Lesions in the nasal region (95.1%; p-value = 0.000) and the pharynx (20.5%; p-value = 0.009) and nasal obstruction (34.4%; p-value = 0.000) were associated with leishmaniasis. Paracoccidioidomycosis was associated with lesions in the oral region (90.4%; p-value = 0.000), oral pain (16.9%; p-value = 0.000), and hoarseness (14.5%; p-value = 0.008). In leishmaniasis, lesions in oral regions were not associated with oral pain and were frequently located close to the nasal area. The manifestations cited above could improve the differential diagnosis of leishmaniasis and paracoccidioidomycosis, and thereby potentially aid in the choice of appropriate confirmatory diagnostic testing.

## Introduction

Leishmaniasis is a parasitic disease caused by species of protozoans of the genus *Leishmania* and transmitted to humans by the bite of phlebotomine sandflies of the genus *Lutzomyia* [1, 2]. The mucocutaneous form in Brazil is mainly associated with the species *L*. *braziliensis* and *L*. *amazonensis* [3]. Paracoccidioidomycosis is a systemic mycosis caused by the fungus *Paracoccidioides brasiliensis* and related species, infecting humans by way of inhalation of its filamentous structures, which are generally deposited in contaminated soil [4, 5]. Mucocutaneous leishmaniasis and paracoccidioidomycosis share similarities in their clinical presentations as infectious diseases with respect to the mucosal lesions [6]. In both diseases, the ulcers may affect the oral, nasal, pharyngeal and laryngeal regions [7]. The injury in mucosa may result in sequelae due to fibrosis, leading to anatomic and functional alterations in the affected sites [4, 8]. A study performed in Brazil indicated that paracoccidioidomycosis was the main differential diagnosis of infectious etiology for leishmaniasis [9]. Therefore, the establishment of a correct and timely diagnosis is essential to avoid complications in both diseases.

Besides the clinical manifestations, leishmaniasis and paracoccidioidomycosis also partially overlap in their territorial distributions [6], with both being endemic in similar regions of Latin America [2, 4]. Health professionals, even in non-endemic countries, must be acutely aware of the differential diagnosis of orofacial lesions, especially because of the increase in travel to endemic areas [10].

Considering the epidemiological and clinical similarities between the diseases, the evaluation of characteristics that support the differential diagnosis between mucocutaneous leishmaniasis and paracoccidioidomycosis has substantial relevance, particularly for the prevention of sequelae. Therefore, this study presents factors that assist in the differentiation of mucosal lesions in these diseases.

## Materials and methods

### Study design

This cross-sectional study included data from medical records of 122 cases of mucocutaneous leishmaniasis and 83 cases of paracoccidioidomycosis attended at the university hospital Cassiano Antonio Moraes (HUCAM), located in Vitória, Espírito Santo State, Brazil. The cases of mucocutaneous leishmaniasis were diagnosed between 1978 and 2009, while the cases of paracoccidioidomycosis were diagnosed between 2001 and 2009.

Inclusion criteria comprised all cases diagnosed as mucocutaneous leishmaniasis or paracoccidioidomycosis with mucosal manifestation in oral, nasal, pharyngeal and laryngeal regions. Exclusion criteria included cases of mucocutaneous leishmaniasis or paracoccidioidomycosis without manifestations in the above-cited regions. The evaluation was performed by the visual direct observation of these regions.

### Definitions

To consider a case of mucocutaneous leishmaniasis confirmed, the parasite or its byproducts should have been detected in tissues and fluids by a direct parasitological examination of either a smear of a scrape obtained from the border of a lesion or an imprint made by the biopsy fragment; by histopathology, or by an isolation in culture [1]. However, since parasites are scarce in the mucosal lesions, indirect methods were also applied for the diagnosis, such as the Montenegro test and the evaluation of successful treatment results [11]. A confirmed case of paracoccidioidomycosis was defined based on the presence of *P*. *brasiliensis* in secretions, body fluid or material from the lesion, detected either by culture or by direct mycological or histopathological exams [4].

Comparison between the cases of mucocutaneous leishmaniasis and paracoccidioidomycosis included the variables sex, age, time of disease evolution (in months), location of the lesion (nasal septum, nasal mucosa, external nose, hard palate, soft palate/uvula/amygdala, tongue, gum, buccal mucosa, lips, pharynx, larynx) and symptoms (oral pain, dysphagia, hoarseness, nasal obstruction).

Time of disease evolution included the time between the emergence of the mucosal lesion and the establishment of diagnosis and was compared between the diseases based on data of cases diagnosed in the 2000’s, in order to avoid differences due to changes in the service access along the decades. Location of the lesion was defined by direct observation, and the symptoms were related to patient complaint.

### Analysis

Statistical analyses were performed with the statistics software R version 3.5.0 [12]. Simple frequencies were calculated for all categorical variables included in the study. Median and interquartile ranges were calculated for age and time of disease evolution. Both variables were compared between mucocutaneous leishmaniasis and paracoccidioidomycosis by means of a Mann-Whitney U Test. Comparisons of the diseases with respect to the dichotomous variables representing the location of the lesion and the symptoms were performed by means of a Pearson Chi-Square Test or a Fisher’s Exact Test. A p-value lower than 0.05 was considered a statistically significant difference between the groups.

### Ethical aspects

The secondary data obtained from medical reports were analyzed anonymously and their use was authorized by the university hospital Cassiano Antonio Moraes (HUCAM). Therefore, no informed consent was obtained from the participants. The research protocol was approved by the Research Ethics Committee of the Health Sciences Center at the Federal University of Espírito Santo (opinion number 190/09).

## Results

Leishmaniasis was diagnosed mainly in adult men. The nasal region presented lesions in 95.1% of cases (n = 116), with the external nose most frequently affected, followed by the nasal septum. Thus, nasal obstruction was the main symptom present in these patients. The oral region had injuries in 28.7% (n = 35) of cases, with the soft palate/uvula/amygdala most frequently affected, followed by the hard palate. No patient with leishmaniasis presented lesions on the tongue or buccal mucosa. Lesions on the pharynx were present in 20.5% of the cases, and the larynx was only affected in 2.5% of the patients. Oral pain, dysphagia, and hoarseness were rare symptoms (Table 1). Time of evolution in leishmaniasis varied along the decades, being this data available for 105 cases (Table 2).

**Table 1:** Comparison of demographic and clinical characteristics in leishmaniasis and paracoccidioidomycosis.

Variable	Leishmaniasis (n = 122)	Paracoccidioidomycosis (n = 83)	p-value
**Male**	88 (72.1%)	76 (91.6%)	0.001
**Age (years)** *Median (Interquartile range)*	52 (37.5–65)	48.5 (43–56)	0.406^#^
**Location of the lesion**			
*Nasal septum*	94 (77%)	1 (1.2%)	0.000*
*Nasal mucosa*	69 (56.6%)	7 (8.4%)	0.000
*External nose*	115 (94.3%)	2 (2.4%)	0.000*
*Soft palate/uvula/amygdala*	31 (25.4%)	25 (30.1%)	0.457
*Hard palate*	13 (10.7%)	19 (22.9%)	0.018
*Gum*	5 (4.1%)	45 (54.2%)	0.000
*Buccal mucosa*	0 (0%)	28 (33.7%)	0.000*
*Tongue*	0 (0%)	8 (9.6%)	0.001*
*Lips*	6 (4.9%)	33 (39.8%)	0.000
*Pharynx*	25 (20.5%)	6 (7.2%)	0.009
*Larynx*	3 (2.5%)	4 (4.8%)	0.444*
**Symptoms**			
*Oral pain*	1 (0.8%)	14 (16.9%)	0.000*
*Dysphagia*	4 (3.3%)	2 (2.4%)	1.000*
*Hoarseness*	5 (4.1%)	12 (14.5%)	0.008
*Nasal obstruction*	42 (34.4%)	0 (0%)	0.000*

^#^ Mann-Whitney U Test

* Fisher’s Exact Test

**Table 2:** Time of disease evolution (months) in leishmaniasis and paracoccidioidomycosis along the decades.

Time of disease evolution	1970’s	1980’s	1990’s	2000’s
**Leishmaniasis**				
*N*	16	26	47	22
*Median (interquartile range)*	48 (5.3–165)	15 (0–63)	24 (6–60)	18 (2–69)
**Paracoccidioidomycosis**				
*N*	0	0	0	57
*Median (interquartile range)*	-	-	-	6 (4–12)

Paracoccidioidomycosis affected mainly adult males. The oral region had injuries in 90.4% of the cases (n = 75), and they were present mainly in the gum, followed by the lips and the buccal mucosa. Thus, oral pain was the primary symptom present in these patients. The nasal region presented lesions in 8.7% of the cases (n = 7), with the nasal mucosa most frequently affected. The pharynx was affected in 7.2% of the cases. Lesions in larynx occurred in 4.8% of the patients, and 14.5% of them presented hoarseness. Dysphagia was a rare symptom, and no patient presented nasal obstruction (Table 1). Data on time of disease evolution for paracoccidioidomycosis was available for 57 cases (Table 2).

Males were the primary group affected by both diseases. Of the two, paracoccidioidomycosis was more strongly associated with this sex, affecting men almost exclusively. Age did not differ significantly between patients of the two diseases. In the 2000’s, the time of evolution was significantly higher in leishmaniasis when compared with paracoccidioidomycosis (p-value = 0.041) (Table 2).

Lesions in the nasal region were significantly associated with leishmaniasis (p-value = 0.000), in addition to lesions in all areas of this site, such as the nasal septum, the nasal mucosa, and the external nose (Table 1). Consequently, nasal obstruction was associated with leishmaniasis (Table 1), especially in patients with lesions in nasal mucosa, as 31 of 69 cases with a lesion in this area had also nasal obstruction (44.9%, p-value = 0.005).

In contrast, paracoccidioidomycosis was significantly associated with lesions in the oral region (p-value = 0.000) and, except for soft palate/uvula/amygdala, lesions in all other areas in this site were associated with paracoccidioidomycosis (Table 1). Accordingly, oral pain was a symptom associated with this disease (Table 1), especially in cases with lesions in hard palate, soft palate/uvula/amygdala and buccal mucosa. Eight of 19 patients with lesions in hard palate had pain (42.1%, p-value = 0.001), as well as eight of 25 of those with lesions in soft palate/uvula/amygdala (32%, p-value = 0.016), and eight of 28 of cases with lesions in buccal mucosa (28.6%, p-value = 0.042). Lesions of the larynx were associated with hoarseness, with three of four patients of paracoccidioidomycosis with injuries in this area presenting the latter (75%, p-value = 0.09).

Soft palate/uvula/amygdala were areas that did not present a significant difference between the diseases (Table 1). However, in leishmaniasis, lesions in this area were associated with a concomitant lesion in the nasal region, with 27 of 31 patients with lesions in the soft palate/uvula/amygdala also having injuries in the nasal region (87.1%, p-value = 0.036). Lesions in the pharynx were associated with leishmaniasis, whereas hoarseness was associated with paracoccidioidomycosis (Table 1). Among leishmaniasis cases with pharynx involvement (n = 25), 92% (n = 23) also presented oral lesions.

## Discussion

In the present study, the demographic profiles of leishmaniasis and paracoccidioidomycosis were similar, with both diseases affecting primarily middle-aged men. In paracoccidioidomycosis, the proportion of males affected was even higher, at a rate of approximately 11 men to one woman, in concordance with the literature [4]. A higher susceptibility to these diseases in males, apart from possible differences in exposure and in risk factors, could be related to the influence of androgen hormones in their pathogenicity, as suggested in previous studies. This could also partially explain a higher occurrence in adults [13, 14]. Furthermore, children and young adults with paracoccidioidomycosis may be more likely to present the acute/subacute form of the disease, which affects mainly the lymphohematopoietic system, with a lower involvement of mucocutaneous regions [15, 16]. Demographic characteristics were similar to those found previously [6], providing further evidence of epidemiological overlap between the diseases, and further underscoring the value of the establishment of clinical parameters for differentiating these two infections.

Leishmaniasis was associated with lesions affecting the nasal region, a common finding in previous studies as well [6, 7, 17, 18, 19]. It appears that a lower temperature in this area favors the local installation of the parasite [3] and diminishes the potential of macrophages to perform phagocytosis [7], contributing to the high frequency of injuries in the external nose. Some studies also link the lesions formation in the nasopharyngeal region in leishmaniosis with the immune response mediated by Toll-like receptors, since the action of some of them may make the host more susceptible to the infection [20–22]. This results in the formation of lesions, some contiguous to the injuries involving cutaneous tissue, and others directly affecting the mucosa [3]. Nasal obstruction is, therefore, a common symptom in these patients [6, 17]; and, in the present study, it occurred mainly in cases with nasal mucosa involvement, suggesting that the proliferation of the lesions in this area has potential to impair the air passage.

The oral region was affected in less than one-third of the cases of leishmaniasis, with lesions present mainly in areas closer to the nasal region, such as the soft palate/uvula/amygdala and the hard palate. Consequently, leishmaniasis lesions were mostly contiguous to the lesions of the nose, as suggested in a previous study [8]. Therefore, evaluation of the nasal region in cases with lesions in the palates can contribute to differential diagnosis, especially when the soft palate/uvula/amygdala is involved, since lesions in these areas have similar frequencies in leishmaniasis and paracoccidioidomycosis. Another important aspect to be evaluated in cases with such lesions is the presence of oral pain, since the report of this symptom was very rare in leishmaniasis. The absence of pain could be partially attributed to the higher time of evolution in patients with leishmaniasis in the 2000’s. However, this hypothesis needs additional research, as there is no data on previous treatment sought by the patients. Previously, studies have identified incorrect diagnoses, such as chronic rhinitis, in cases of leishmaniasis presenting nasal obstruction [18]; and, especially in cases with unusual manifestations, such as lips single lesion [10], there were a high number of delayed correct diagnoses, resulting in the worsening of the lesions over time [23]. Lesions in the pharynx were associated with leishmaniasis, being present in almost one-quarter of the cases, and, in the majority of them, with concomitant oral involvement, as described in a previous study [8].

In contrast, paracoccidioidomycosis was associated with lesions in the oral region and oral pain, especially when hard palate, soft palate/uvula/amygdala, and buccal mucosa were involved. Lesions in oral mucosa [6, 15, 24, 25–29] and oral pain were also reported previously in separate studies [6, 25, 29, 30]. Pain during chewing and during the practice of oral hygiene negatively impacts the patient’s state of overall health, as it contributes to the depletion of nutrients [31]. Weight loss is a common occurrence in paracoccidioidomycosis, especially in cases where the lungs are involved [4], and may be due in part to decreased eating due to discomfort. Trauma to tissue during mastication could be associated with the location of the injuries in paracoccidioidomycosis, as they are more common in gum, lips, and buccal mucosa, as demonstrated here and in previous studies [24, 27, 29]. Accordingly, lesions in the oral region were asymmetric, as opposed to the symmetric lesions found in leishmaniasis. In paracoccidioidomycosis, the sites of masticatory traumas determine the locations of injuries, whereas, in leishmaniasis, the location of the injuries is associated with areas of lower temperatures due to the passage of air. Hoarseness was associated with paracoccidioidomycosis, being present in almost all cases with lesions in the larynx. The number of patients whose larynx was affected could be underestimated in the present study, considering that hoarseness is generally associated with lesions in this site; therefore, the actual frequency of injuries to the larynx might have been higher than what was reported. Compared to a previous investigation [6], the presence of lesions in the larynx was lower in the present study, which may be due to lack of examination of this area.

This study exhibits some limitations inherent to the use of secondary data. Therefore, imprecisions in the reports of signs and symptoms could be present in the medical records. The assumption on limited external validity due to the inclusion of institutional-based cases may be mitigated since HUCAM was the reference center up to 2010 for diagnosis of both diseases in the Espírito Santo State and the bordering municipalities of neighboring states, from where the cases were referenced, even when they were non-severe, being mostly attended in the outpatient regimen. The difference in the distribution of the diseases along the decades could result in variations in the time of disease evolution due to changes in the service access. Nevertheless, when comparing the diseases in the same decade, differences in the time of evolution are evident. For the other variables, since no changes occurred on the diagnostic criteria, technologies applied in the clinical evaluation, and in the medical team involved in the cases conduction, possibly there is no significant influence of distinct decades in the results.

Even with the above-cited limitations, the study presents important aspects to be evaluated in the differential diagnosis of leishmaniasis and paracoccidioidomycosis. Successful differential diagnosis of these diseases can increase the precision of the indication of diagnostic tests, thus reducing medical costs. In addition, this study is unique in the congregation of a high number of reports of both diseases in a similar region, in part due to the distribution and concentration of them in few geographic areas localized especially in Brazil, leading to the lack of publications evaluating clinical aspects of both diseases in other countries. Therefore, additional studies in other places including infectious diseases with similar mucosal manifestations are needed, in order to further improve the accuracy and expedience of the management of patient care.

## Supporting information

S1 File Dataset. (XLSX)
